# Spleen-Dependent Regulation of Antigenic Variation in Malaria Parasites: *Plasmodium knowlesi SICAvar* Expression Profiles in Splenic and Asplenic Hosts

**DOI:** 10.1371/journal.pone.0078014

**Published:** 2013-10-18

**Authors:** Stacey A. Lapp, Cindy Korir-Morrison, Jianlin Jiang, Yaohui Bai, Vladimir Corredor, Mary R. Galinski

**Affiliations:** 1 Emory Vaccine Center, Yerkes National Primate Research Center, Emory University, Atlanta, Georgia, United States of America; 2 Departamento de Salud Pública, Facultad de Medicina, Universidad Nacional de Colombia, Bogotá, Colombia; 3 Department of Medicine, Division of Infectious Diseases, Emory University, Atlanta, Georgia, United States of America; University of Copenhagen, Denmark

## Abstract

**Background:**

Antigenic variation by malaria parasites was first described in *Plasmodium knowlesi*, which infects humans and macaque monkeys, and subsequently in *P. falciparum*, the most virulent human parasite. The schizont-infected cell agglutination (SICA) variant proteins encoded by the *SICAvar* multigene family in *P. knowlesi*, and Erythrocyte Membrane Protein-1 (EMP-1) antigens encoded by the *var* multigene family in *P. falciparum*, are expressed at the surface of infected erythrocytes, are associated with virulence, and serve as determinants of naturally acquired immunity. A parental *P. knowlesi* clone, Pk1(A+), and a related progeny clone, Pk1(B+)1+, derived by an *in* *vivo* induced variant antigen switch, were defined by the expression of distinct SICA variant protein doublets of 210/190 and 205/200 kDa, respectively. Passage of SICA[+] infected erythrocytes through splenectomized rhesus monkeys results in the SICA[-] phenotype, defined by the lack of surface expression and agglutination with variant specific antisera.

**Principal Findings:**

We have investigated *SICAvar* RNA and protein expression in Pk1(A+), Pk1(B+)1+, and SICA[-] parasites. The Pk1(A+) and Pk1(B+)1+ parasites express different distinct *SICAvar* transcript and protein repertoires. By comparison, SICA[-] parasites are characterized by a vast reduction in *SICAvar* RNA expression, the lack of full-length *SICAvar* transcript signals on northern blots, and correspondingly, the absence of any SICA protein detected by mass spectrometry.

**Significance:**

SICA protein expression may be under transcriptional as well as post-transcriptional control, and we show for the first time that the spleen, an organ central to blood-stage immunity in malaria, exerts an influence on these processes. Furthermore, proteomics has enabled the first in-depth characterization of SICA[+] protein phenotypes and we show that the *in* *vivo* switch from Pk1(A+) to Pk1(B+)1+ parasites resulted in a complete change in SICA profiles. These results emphasize the importance of studying antigenic variation in the context of the host environment.

## Introduction

Antigenic variation in malaria entails the sequential expression of different high molecular weight parasite-encoded variant proteins from a large multigene family on the surface of infected red blood cells (iRBCs) and this process can result in immune evasion and facilitate disease, reviewed in 1–3. In the original observations made by Brown and Brown in 1965, successive waves of *P. knowlesi* parasitemia in rhesus macaques exhibited distinct antigenic profiles, as based on the agglutination of schizont-infected erythrocytes [4] indicating that immunogenic targets on the surface of the infected erythrocytes changed antigenically during the course of an infection [5]. These variant antigens were named for the assay that detected them, Schizont Infected Cell Agglutination (SICA) antigens. Specific SICA agglutinating and opsonizing antibodies were shown to factor into proposed mechanisms of immunity being raged to fight the infection in these monkey hosts [6,7]. A series of phenotypically distinct *P. knowlesi* SICA[+] clones were subsequently generated by the *ex vivo* micromanipulation of single schizont iRBC to infect monkeys and effect their *in vivo* propagation in naïve animals [8,9]. When passaged in pre-exposed/immunologically boosted rhesus macaques, a switch in variant antigen expression was observed; newly developed clones derived therefrom had unique iRBC surface antigenic properties [8,9]. These clones were key to demonstrate the clonal nature of the antigenic variation and they enabled the experimental identification of the SICA variant antigens as high molecular weight (~200 kDa) parasite-encoded proteins that were extractable in SDS, and predicted to be inserted in the membrane of the RBC with exposure on the surface of the infected cells [8-13]. The analogous protein in *P. falciparum* (Erythrocyte Membrane Protein 1; PfEMP1) was identified based on similar procedures and considerations [14,15]. PfEMP1 is responsible not only for antigenic variation in *P. falciparum* reviewed in 2,3 but the cytoadherence and sequestration of *P. falciparum* iRBCs [16] and associated malarial pathogenesis [1,2,17-19]. Sequestration is also known to occur in *P. knowlesi*, but to a much lesser degree and in different tissue locations [20].

In splenectomized rhesus macaques, *P. knowlesi* iRBCs lose expression of the SICA variant antigens at the surface and concomitantly result in less virulent infections that are readily controlled in spleen-intact rhesus macaques if SICA is not expressed [9,21,22]. If these parasites (called SICA[-]) re-express the SICA antigen at the surface of the host RBCs when returned to an intact animal, they regain a high level of virulence that can result in an overwhelming parasitemia and death of the animal if not treated [9]. These observations bring emphasis to the importance of the spleen in regulating variant antigen expression, yet the mechanism(s) involved has remained unknown. The original studies also did not address whether the SICA protein was produced but not transported to the surface of the infected host cells, or not produced at all. Other studies performed with *P. falciparum* in intact or splenectomized *Saimiri sciureus* monkeys also demonstrated that the spleen modulates the expression of the variant antigens in this species also, which are associated with cytoadherence and sequestration [23,24]. A few clinical case studies with *P. falciparum* infections in splenectomized humans also indicate that in the absence of a spleen the cytoadherent properties of iRBCs largely disappear, which would correspond to the down regulation of the variant antigens that are involved in the cellular adhesion and sequestration of *P. falciparum* iRBCs [25,26], and reviewed in 27. Another analysis from fatal cases of *P. falciparum* evaluates the potential immunological host-parasite interactions within the spleen involving trophozoite or schizont infected RBCs [28].

The *P. knowlesi* SICA[+] clone denoted as Pk1(A+) is the parent of the Pk1(B+)1+ clone [8,9] derived through an *in vivo* conducted antigen switch and recloning. The Pk1(A+) and Pk1(B+)1+ parasite clones (and their SICA[-] progeny) share the same genotype; the only genetic difference known so far is due to a mitotic recombination event that resulted in distinctly different cytoplasmic domains and 3’UTRs for the respective 205A and 205B *SICAvar* gene alleles [29,30]. The *in vivo* induced switch from the Pk1(A+) to Pk1(B+)1+ phenotype was the direct result of the parasite’s ability to change in the face of an immune response against the SICA antigens expressed at the surface of the Pk1(A+) iRBCs [8,9]. Antisera raised against the Pk1(A+) iRBCs did not crossreact with the Pk1(B+)1+ iRBCs and vice versa. Using both metabolic and surface radioiodination labeling methods, these clones were shown on SDS-PAGE gels to express distinct SICA protein doublets of 210/190 kDa and 205/200 kDa, respectively [8]; a few minor clone-specific bands were also observed that differed between the clones and depending on the labeling method [8,10]. After many short-term (< 1 week) passages propagating the parasite in naïve rhesus monkeys, typically done to maintain long-term frozen stocks, the respective clonal populations maintained the expression of the major protein phenotypes [29,30]. Whether the two major protein bands of each phenotype represent two distinct proteins or processed products, whether other minor protein bands observed corresponded to related or novel variant proteins, and whether they were in fact co-expressed on each iRBC, remained open questions [8,10]. When the first *SICAvar* gene was identified and characterized, subtractive immunoprecipitation experiments with specific antibody reagents supported the view that the two protein bands represented two distinct proteins [29]. Importantly, related SICA[-] clones denoted as Pk1(A-)1- and Pk1(B-)1-, created from passage in splenectomized rhesus macaques, did not express these antigens at the surface of the iRBCs, as determined by agglutination assays and comparable radiolabelled immunoprecipitation experiments [9].

The gene encoding the 205 kDa SICA protein expressed by the Pk1(B+)1+ parasites was reported in 1999 with the discovery of the *SICAvar* multigene gene family [29]. This report described the complex multi-exon *SICAvar* gene structure with extensive diversity in the context of seven cysteine-rich domains and a conserved cytoplasmic domain encoded by the final exon [29]. With the advent of the *P. knowlesi* genome sequencing project, data mining with a special focus on the conserved cytoplasmic domain encoding regions and the 3’UTRs predicted 108 *SICAvar* genes and defined elaborate conserved 3’UTR sequences with sequential ‘Blocks’ defined by repeats (Block I), A/T homopolymers (Block II), and a GC rich-domain (Block III) [30]. These studies confirmed that *SICAvar* transcripts encoding 205 alleles (among others) were produced in both the Pk1(A+) and Pk1(B+)1+ iRBCs, despite that lack of expression of the 205kDa protein in the Pk1(A+) parasites; qRT-PCR data suggested that a processing event between Blocks II and III may have resulted in the inactivation of the *205A* allele transcript [30]. The *SICAvar* gene encoding the 205 kDa SICA protein was originally defined by a 10-exon structure within an approximate 14 kb segment of genomic DNA, and this was recently redefined with a total of 12 exons. The redefined gene was shown to be centrally located on chromosome 5 and includes the true 5’UTR and two distant upstream exons, separated from the original 10-exon sequence by a large 12 kb repeat-laden intron [31]; such large upstream introns are common to a subset of *SICAvar* genes [31,32]. The *P. falciparum* *var* genes in stark contrast have evolved to have only two exons. The expressed proteins of the *SICAvar* and comparable *var* gene families from *P. falciparum* are biochemically and functionally analogous as variant antigens [8,15,33], and shown to be related by proteomics [34], despite a high degree of inherent diversity. A recent evolutionary analysis of these and other multigene families across species also supports the ancestral relationship of *SICAvar* and *var* genes [35]. The first exon of *var* genes encodes protein sequences that represent the external part of the variant antigens, comparable to all but the final exon of the *SICAvar* genes: in both species the bulk of each variant antigen gene encodes a series of cysteine-rich domains followed by a transmembrane domain in the penultimate exon and the final exon encodes a highly conserved cytoplasmic domain [1,19,29,31,36-38]. 

Continued investigation of the structure and expression of the *SICAvar* gene family in parallel with the *var* gene family is important for studying the mechanism of antigenic variation in the context of the host, but this has been a challenge, despite the publication of the first *P. knowlesi* genome sequence [32]. This is because of the complex multi exon-intron structures, extensive homology amidst diversity in the exons, many large A-T rich introns, and UTRs with extensive repeat sequences [29-32]. All of these factors have made it difficult to accurately link the coding and non-coding sequences comprising each gene and appropriately assign these genes to the parasite’s 14 chromosomes in order to study the gene expression of this family. A majority of the *SICAvar* genes in fact currently remain in fragments in the *P. knowlesi* database (222 out of the 244 annotated *SICAvar* and *SICAvar*-like sequences) including the prototype 12-exon *SICAvar* gene described above [29-31]. Therefore, at this stage, traditional gene-walking methods must still be relied on to verify data coming from post-genomics research, including proteomics, as reported here. 

The overall aim of this study was to understand the basic molecular definition of SICA[-] parasites generated in splenectomized rhesus monkeys with regards to the expression of the *SICAvar* gene family (based on the Pk1(A-)1- and Pk1(B-)1- cloned populations), and develop complementary baseline SICA[+] proteomes for the Pk1(A+) and Pk1(B+)1+ clonal populations to support investigations on the mechanism of variant antigen switch events. This study shows that *SICAvar* genes are transcribed in SICA[-] parasites, though at a much lower level compared to SICA[+] parasites, as evaluated by quantitative RT-PCR. Of particular importance, full-length *SICAvar* transcripts were not evident in SICA[-] RNA on northern blots, and SICA protein was not detected in SICA[-] iRBCs by Liquid Chromatography Tandem Mass Spectrometry (LC-MS/MS). We have concluded that in the absence of the spleen, there is a complete downregulation of SICA protein expression, which is dependent on some combination of *SICAvar* transcription regulatory mechanisms and possible post-transcriptional control processes; antisense transcripts may also play a role. Moreover, we have classified the Pk1(A+) and Pk1(B+)1+ clonal populations by distinct repertoires of SICA antigens, with apparent dominant and minor expressed proteins. This knowledge provides a new starting point for in depth studies on the molecular mechanisms that govern the genetic and epigenetic expression and switching of *P. knowlesi* variant antigen genes, transcripts and proteins *in vivo* and future comparative studies with *P. falciparum*.

## Results

### SICA proteins are not detected in SICA[-] parasites

Specific antibodies to any region of the SICA proteins were not available at the time when SICA[-] parasites were first identified, and thus it has remained an open question whether these parasites do not make any protein or whether it is produced but not transported to the surface of the iRBCs [9,21,22]. To answer this question we made use of specific antiserum generated against the conserved final exon cytoplasmic domain encoding sequences from the *205B SICAvar* allele [29]. BLAST searches using this sequence had predicted 108 *SICAvar* genes in the *P. knowlesi* genome [30] and the antiserum was predicted to react with all expressed SICA proteins. When tested by IFA, the pan-SICACyto antiserum was positive as expected on all SICA[+] trophozoite-infected RBCs but negative on SICA[-] trophozoite-infected RBCs with only an occasional, spurious fluorescing iRBC observed in the blood smears (not shown). These data were corroborated by the absence of SICA protein detection by immunoprecipitation and LC-MS/MS experiments. 

**Figure 1:**
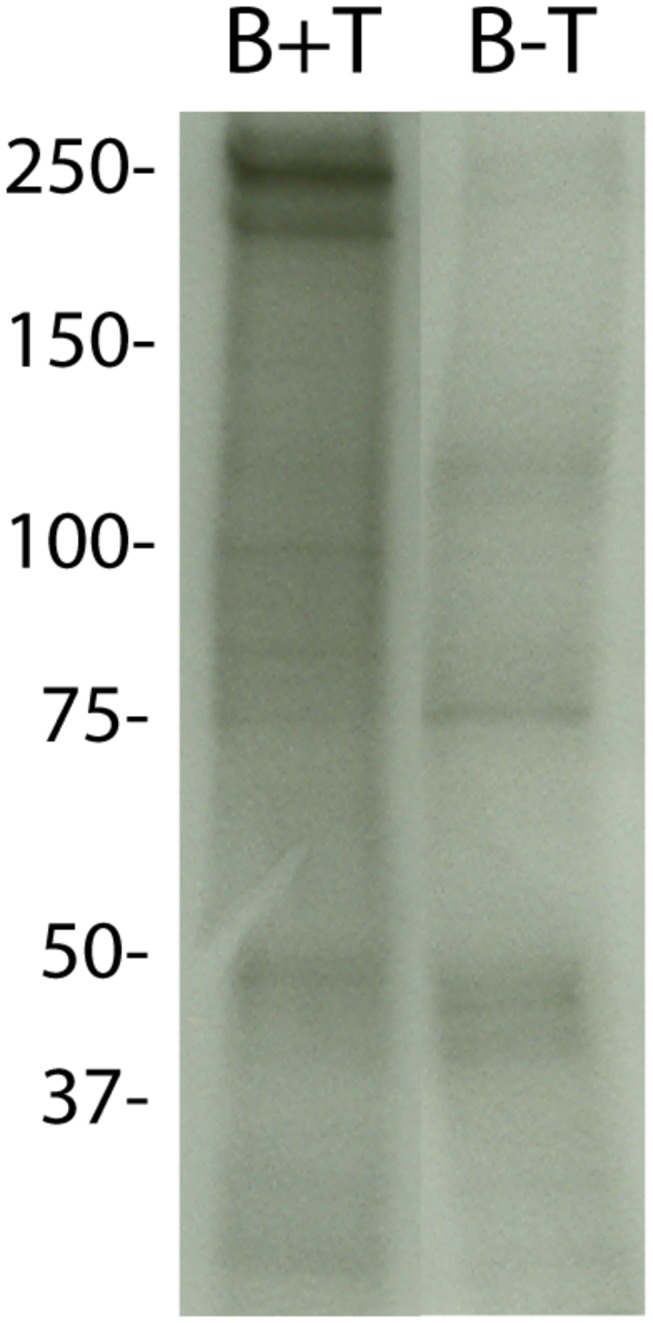
SICA protein profile from Pk1(B+)1+ and Pk1(B-)1- immunoprecipitations. Trophozoite SDS extracts were immunoprecipitated with purified IgG from an antiserum (Cyto17) (Al-Khedery, 1999) that is specific for the SICA conserved cytoplasmic domain. The intense bands visible around 250 kD in the Pk1(B+)1+ parasites but lacking in the Pk1(B-)1- clone represent the SICA proteins.

Initially, extracts of metabolically labeled Pk1(B+)1+ and Pk1(B-)1- late-stage trophozoites were immunoprecipitated with IgG antibodies purified from another antiserum raised against the *205B SICAvar* allele cytoplasmic domain, called Cyto17 [29]. High molecular weight SICA proteins in the 200 kDa range were evident in the Pk1(B+)1+ parasites, but not in the Pk1(B-)1- parasites (Figure 1). These studies focused on the Pk1(B+)1+ and the Pk1(B-)1- clones, since only the gene encoding the 205 kDa SICA protein characteristic of the Pk1(B+)1+ parasites had been confirmed and reported in the in the literature [29-31]. In pilot experiments based on less sensitive MS/MS methods, SICA protein was likewise not detected from either Pk1(A-)1- or Pk1(B-)1- samples. In these studies, whole trophozoite extracts and purified trophozoite-infected RBC membranes were separated by SDS-PAGE followed by gel extraction, trypsin treatment of Coomassie-stained bands, and tandem MS analysis, as performed in previous proteomic investigations of SICA and its relationship with PfEMP1 [34]. In contrast to the SICA[+] samples, showing positive hits to *SICAvar* gene identifications (IDs), no significant hits to *SICAvar* gene IDs were obtained from any SICA[-] samples. The direct approach of immunoprecipitating SICA proteins prior to LC-MS/MS analysis was undertaken to best ensure the detection of any SICA proteins in these parasites, knowing that SICA proteins are not abundant at the surface of infected host cells and proteins expressed at low levels could go undetected if masked by other more abundant proteins. When immunoprecipitates from either Pk1(A-)1- or Pk1(B-)1- parasites were subjected to LC-MS/MS analysis, no SICA peptides were identified.

### *SICAvar* full-length transcripts are detected by northern blot analysis in SICA[+] but not SICA[-] parasites

**Figure 2:**
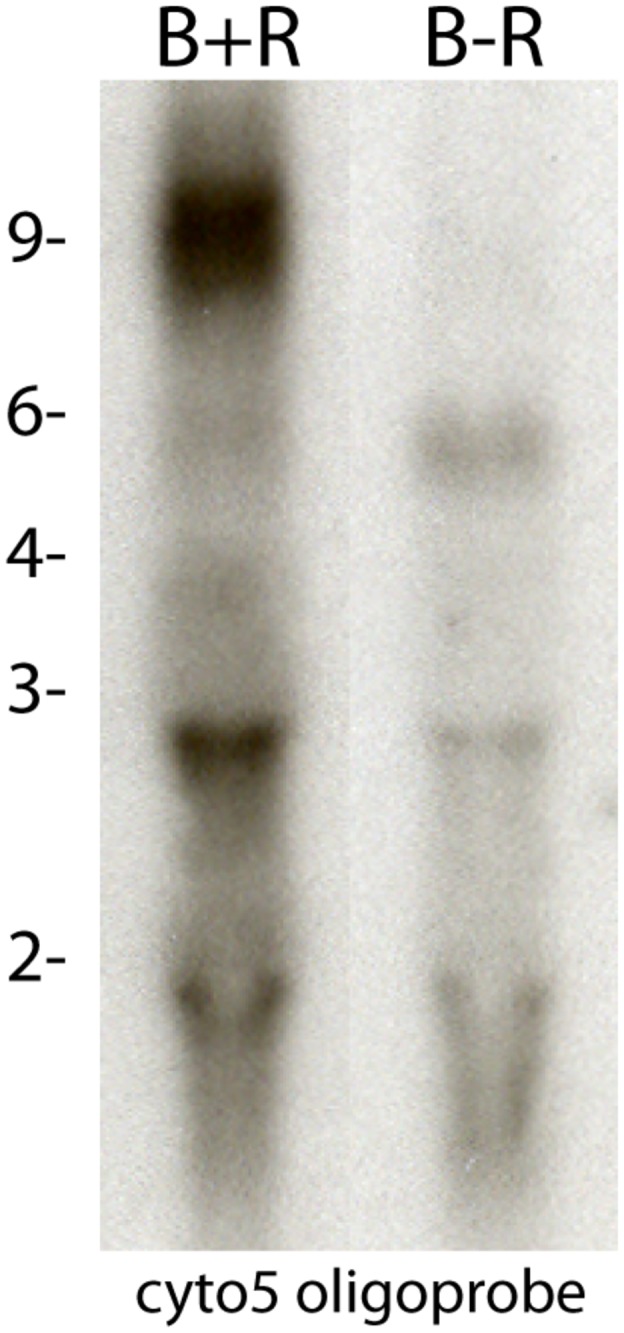
Northern blot of Pk1(B+)1+ and Pk1(B-)1- ring-stage RNA. This is evidence that *SICAvar* transcript regulation differs in SICA[+] compared to SICA[-] parasites, raising questions regarding what transcriptional and post-transcriptional gene expression mechanisms come into play depending on whether a host is intact or splenectomized. Pk1(B+)1+ and Pk1(B-)1- ring-stage total RNA was probed with an antisense oligoprobe representing the highly conserved cytoplasmic-encoding final exon of all *SICAvar* genes.

We likewise examined *SICAvar* gene expression in SICA[+] and SICA[-] parasites by focusing on the Pk1(B+)1+ and related Pk1(B-)1- clones. The 205 kDa SICA protein is encoded by the prototype 12-exon *SICAvar* gene and a large transcript of ~9 kb [31]. The Pk1(B+)1+ and Pk1(B-)1- ring-stage total RNA was probed with an antisense oligoprobe (Cyto5) designed from the highly conserved cytoplasmic-encoding final exon of *SICAvar* genes (Figure 2). *SICAvar* transcript signals of the expected size were apparent in the Pk1(B+)1+ RNA, but not in the Pk1(B-)1- RNA. Comparable data was also generated with larger cytoplasmic domain probes that gave very intense signals on the SICA[+] samples (not shown) and when evaluating *SICAvar* expression in the Pk1(A+) and Pk1(A-)1- clones (discussed below). 

### Quantification of *SICAvar* transcripts in Pk1(B+)1+ and Pk1(B-)1- RNA

**Figure 3:**
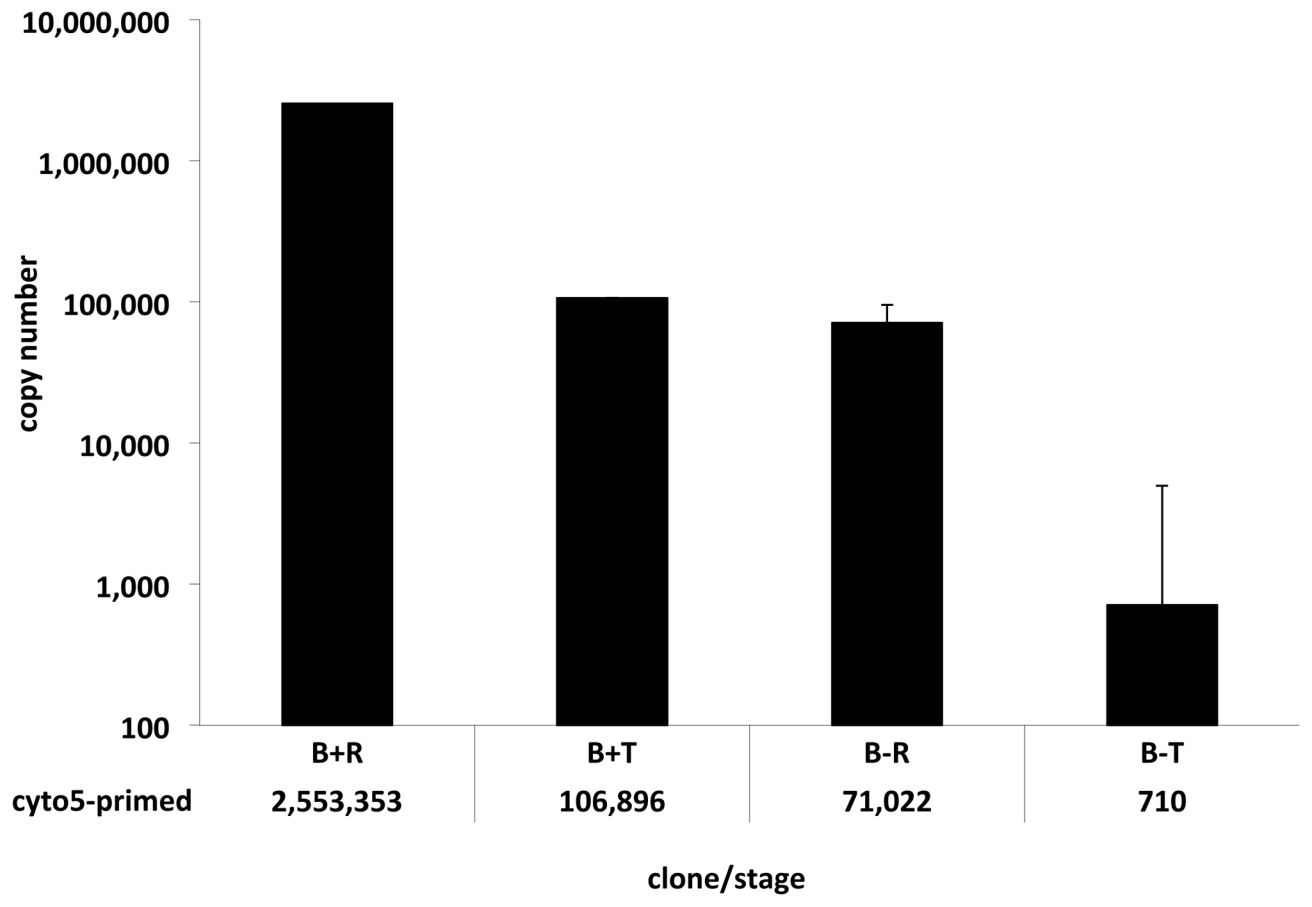
Quantitative Real Time-PCR of the 205B allele in Pk1(B+)1+ and Pk1(B-)1- ring and trophozoite stage parasites. Copy number profiles showing that *SICAvar* transcripts representing the 205 kDa SICA protein that is expressed by the Pk1(B+)1+ parasites are most abundant in the ring stage (R) and reduced in the trophozoite stage (T), while greatly reduced in comparable parasite samples from the Pk1(B-)1- clone. The conserved exon 12 based Cyto5 primer was used to initiate the experiment.

Quantitative RT-PCR experiments were then designed to amplify a specific region of the *SICAvar* transcript encoding the 205 kDa SICA protein from the Pk1(B+)1+ parasites (Figure 3); the amplicon corresponds to a 100 bp region within the penultimate exon, just upstream of the Cyto5 sequence encoded by the highly conserved final *SICAvar* exon (Table S1). In pilot experiments, a marked difference was observed in transcript number between cDNA that was primed with oligo(dT) versus the Cyto5 oligonucleotide, consistent with the fact that small stretches of adjacent adenosine residues in the Block II region of the 3’ UTR can serve as binding sites for oligo(dT) and cause a misrepresentation of transcript number [30]. We therefore used Cyto5-primed cDNA in qRT-PCR analyses aiming to quantify *SICAvar* transcripts.

The *205B SICAvar* transcripts were detected in the Pk1(B+)1+ clone, as expected (Figure 3). The *205B SICAvar* transcript copy number (2,553,353 compared to 106,896) was 24-fold greater in the ring-stage RNA versus the trophozoite-stage RNA, consistent with general expectations based on stage-specific northern blot data and original qRT-PCR [29,30]. In stark contrast, when comparing the ring-stage RNA from Pk1(B+)1+ and Pk1(B-)1- parasites, the amplified *205B SICAvar* sequences detected were 36-fold less in the Pk1(B-)1- samples, with respective copy numbers of 2,553,353 compared to 71,022. Comparisons of the *205B SICAvar* copy numbers detected in the Pk1(B-)1- ring and trophozoite-stage RNA samples showed an additional 100-fold difference (71,022 versus 710 copy numbers) (Figure 2). These data indicate that transcripts are being made in the SICA[-] parasites, but at a much lower level than in the SICA[+] parasites, and that they are more quickly eliminated. Antisense *SICAvar* RNA was also detected by northern blot, qRT-PCR and RNAseq experiments (not shown), but its mechanistic relevance, if any, remains uncharacterized at this time; further studies to explore the meaning of such transcripts will benefit from the improved genome sequence and RNAseq data that is in progress (unpublished data).

### Distinct sets of predominant and minor SICA proteins are detected by LC-MS/MS in the Pk1(A+) and Pk1(B+)1+ parasite cloned populations

**Figure 4:**
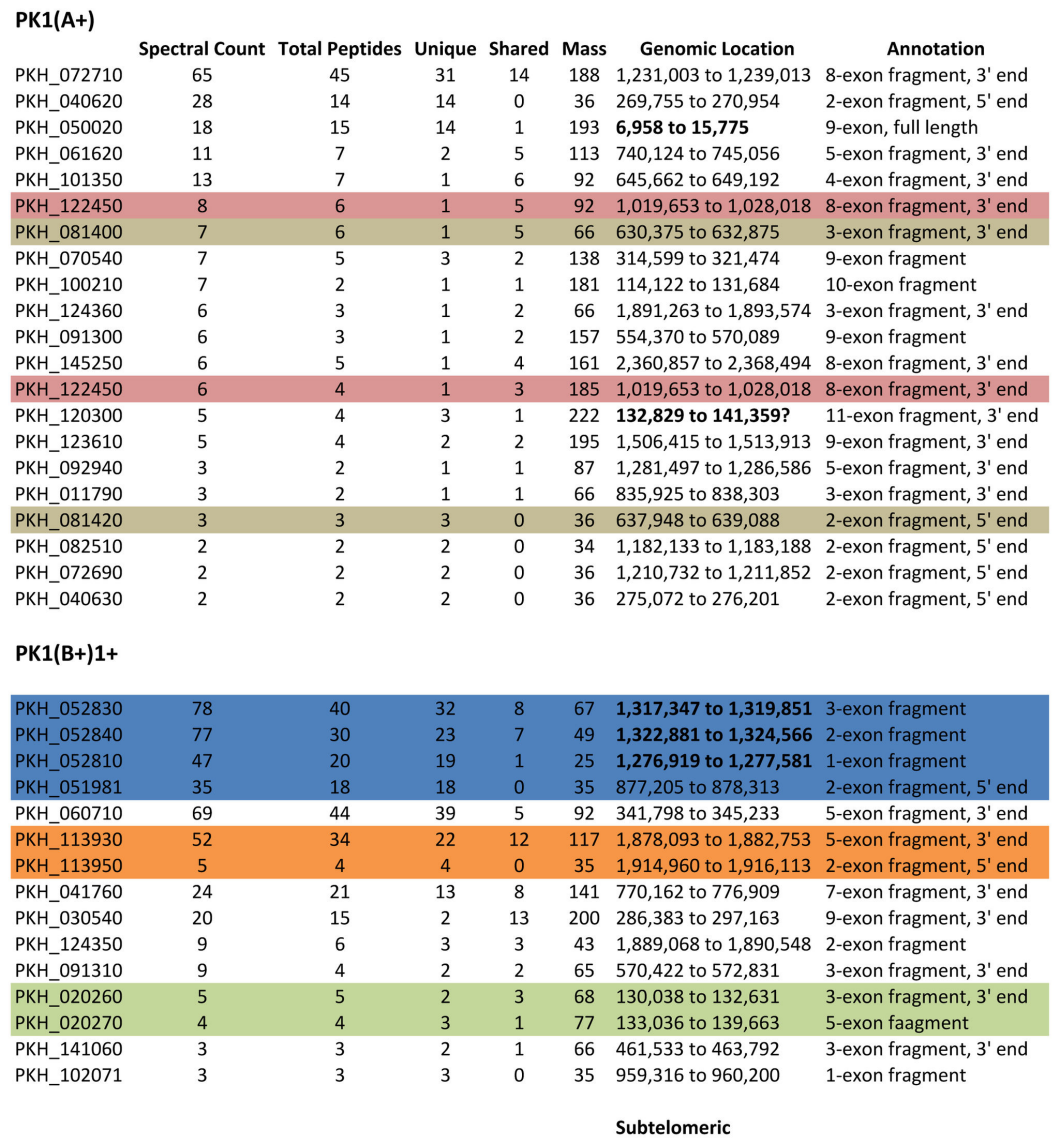
Table listing *SICAvar* gene IDs determined by LC-MS/MS analysis of Pk1(A+), Pk1(B+)1+ parasites. Immunoprecipitates were generated with a pan-SICACyto antibody and trophozoite extracts from Pk1(A+), Pk1(B+)1+ protein expression. Gene IDs representing the same gene are similarly color-coded. Subtelomeric genes are in bold.

**Figure 5:**
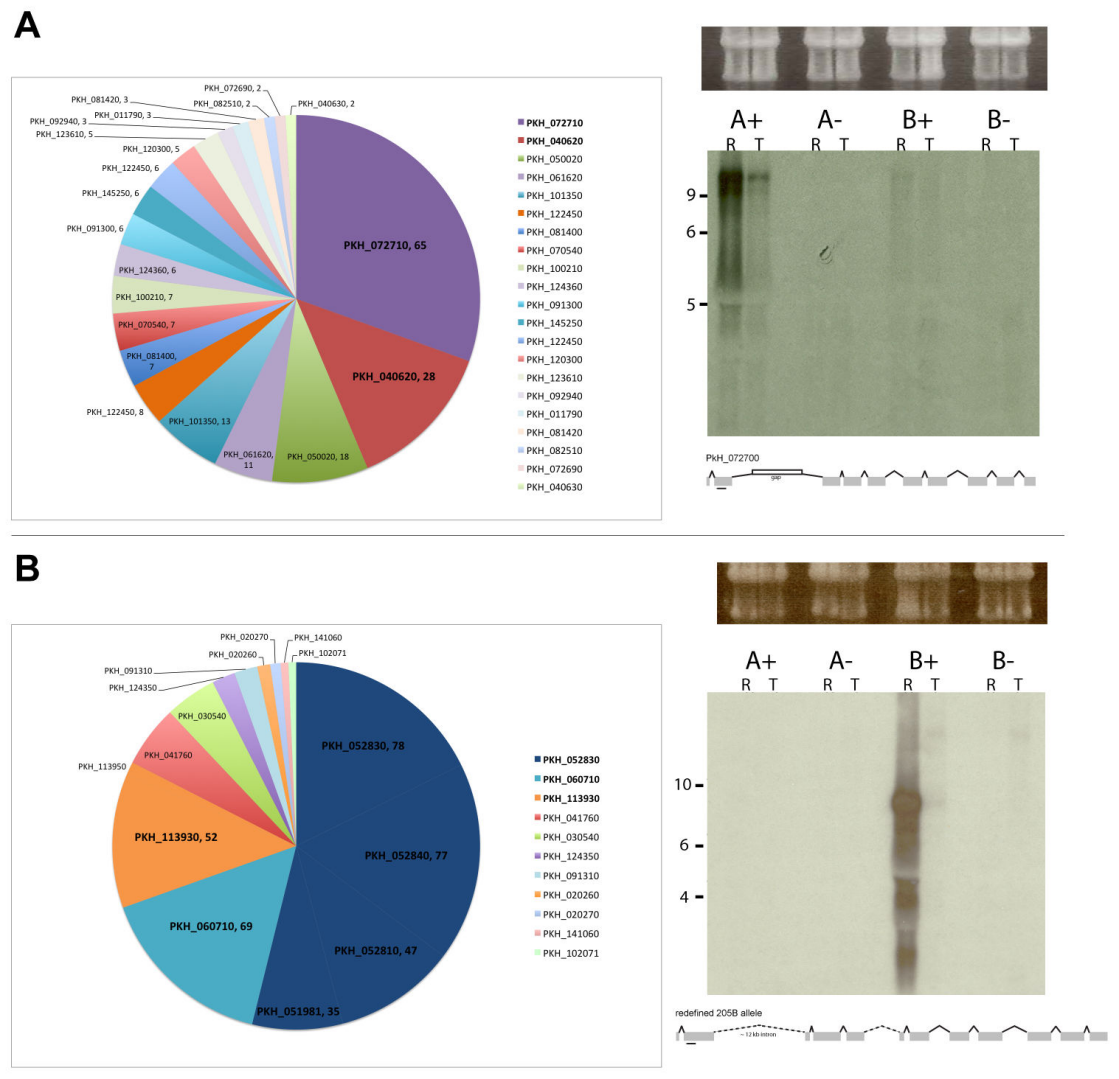
Relative abundance of peptide hits by spectral count and transcripts by northern blot of Pk1(A+) and Pk1(B+)1+ parasites. (A) Pie charts depicting the SICA protein expression profiles shown in Figure 4, with relative abundance indicated by spectral counts. (B) Northern blots of ring and trophozoite stage RNA from the Pk1(A+) and Pk1(B+)1+ clones and their respective SICA[-] clones hybridized to gene-specific antisense riboprobes representing the most dominantly expressed SICA protein shown in the Table and Pie Chart. Schematics of known information on these *SICAvar* genes and the location of the riboprobes are shown by an underline. Total RNA ethidium bromide stain is shown above the respective blot.

Proteomic approaches were also used to determine which *SICAvar* genes were expressed as proteins in the Pk1(A+) and Pk1(B+)1+ trophozoite-infected RBCs, in addition to the *SICAvar* gene known to encode the 205 kDa SICA protein expressed by the Pk1(B+)1+ parasites [29,31]. The pan-SICACyto antiserum was used to immunoprecipitate any SICA proteins present in extracts of the Pk1(A+) and Pk1(B+)1+ trophozoite-infected RBCs. LC-MS/MS analyses were performed to assess the immunoprecipitated proteins and distinct SICA expression profiles were obtained for each clone (Figures 4, 5A and 5B). Strikingly, these data reflect a complete change in the repertoire of expressed SICA proteins in the trophozoite-infected RBCs of the Pk1(A+) and switched Pk1(B+)1+ phenotype. It is also important to recognize that the published genome database with 8X coverage [32] overall was in reasonable shape for initiating these studies, but any *SICAvar* genes that remain fragmented in the database or not yet assigned to chromosomes may be underrepresented in the proteomic profiles. Figure 4 also shows the status of the *SICAvar* gene sequences in the published genome database (complete or incomplete) corresponding to the various gene IDs detected.

The predominant SICA protein identified in the Pk1(A+) trophozoite-iRBCs corresponds to the gene ID PkH_072710; relative abundance was based on the spectral count (Figures 4 and 5A). This ID represents an 8-exon *SICAvar* fragment, which includes the final conserved exon that encodes the protein’s cytoplasmic domain and seven upstream exons. There is a sequence gap immediately upstream of this 8-exon fragment, which is followed by gene ID PkH_072700: with a PEXEL-containing, 2-exon *SICAvar* fragment. As in our previous investigations redefining the *205B SICAvar* allele [31], we confirmed the continuity of these sequences by RT-PCR. Thus, we report here that the predominantly expressed Pk1(A+) *SICAvar* mRNA transcript is encoded in a 10-exon *SICAvar* gene, represented in the published genome by neighboring gene IDs PkH_072700 and PkH_072710 (Figure 5A). 

Another gene fragment, PkH_040620, corresponded to the second most abundant SICA protein in the Pk1(A+) trophozoite-infected RBCs. This gene ID is currently annotated as a PEXEL-containing 2-exon *SICAvar* sequence with a gap immediately downstream and no additional *SICAvar* exons following the gap, in contrast to the instance noted above. We therefore have concluded that the rest of this *SICAvar* gene sequence is missing from the current genome assembly [32], similar to another sequence we reported previously as missing (corresponding to the 3’ region of the *205B SICAvar* allele, [31]). We therefore have no means to determine the identity of the remainder of this gene at this time and we cannot complete the sequence by gene-walking methods. Still, based only on the initial 2-exon sequence, the LC-MS/MS generated BLAST search identified the PkH_040620 encoded SICA protein as a predominant hit. If this entire multi-exon *SICAvar* sequence were present in the genome database, we predict that this protein would have been recognized in even greater abundance, and in turn represented by a larger segment in the pie chart, perhaps comparable to ID PkH_072710 (Figure 5A). Several other SICA proteins were identified in the Pk1(A+) trophozoite-infected RBCs at lower levels (Figure 4 and 5A). Among these, curiously, PkH_040630, detected with two unique peptides, represents an isolated two-exon sequence with a PEXEL motif and apparent start and stop codons. This gene ID is immediately upstream from the above-mentioned gene ID PkH_040620.

The SICA protein that is encoded by the originally characterized *205B SICAvar* allele [29-31] was confirmed in the Pk1(B+)1+ trophozoite-infected RBCs as being the most abundant (Figure 4 and 5B); corroborating the original data identifying this gene as the one that encodes the 205 kDa SICA protein that is characteristic of the Pk1(B+)1+ clone [8]. Although fully characterized in the original reports [29-31], this gene remains annotated in the published *P. knowlesi* genome database as five independent and disjointed *SICAvar* fragments [32]. Regardless, proteomic analysis resulted in the positive identification of this gene, with hits to four of the fragments spanning the N-terminal, middle, and C-terminal regions. 

Two additional SICA proteins were identified in the Pk1(B+)1+ trophozoite-infected RBCs with comparatively high spectral counts: 69 and 52, corresponding to gene IDs PkH_060710 and PkH_113950, respectively; gene fragment PkH_113930 was also identified and we confirmed by RT-PCR that its encoded sequence in the amplified message is contiguous with the encoded sequence of PkH_113950. PkH_060710 is annotated as a 5-exon *SICAvar* fragment, lacking its 3’ end, with an apparent large gap in the genome assembly immediately downstream from this sequence. Several BLAST searches of shotgun data using available sequences from GeneDB did not reveal any additional downstream sequence for this gene, and RT-PCR amplifications using conserved and degenerate primers recognizing the 3’ region of PkH_060710 and downstream unannotated ORF did not result in the linkage of these sequences. Several other SICA proteins were detected in the Pk1(B+)1+ trophozoite-infected RBCs at a lower level, including PkH_041760 and PkH_030540, with spectral counts of 24 and 20, respectively (Figure 5B). Notably, the *SICAvar* genes identified in these analyses map to loci throughout all chromosomes (Figure S1). 

### Northern blots confirm the ring-stage expression and maintenance of distinct full-length *SICAvar* transcripts representing predominant proteins in the Pk1(A+) and Pk1(B+)1+ infected RBCs, and their absence in the Pk1(A-)1- and Pk1(B-)1- RNAs

Northern blot experiments were carried out with Pk1(A+), Pk1(A-)1-, Pk1(B+)1+ and Pk1(B-)1- total RNA samples from the ring and trophozoite stages of development and hybridized with probes representing the most predominant SICA detected by LC-MS/MS in the Pk1(A+) and Pk1(B+)1+ trophozoite- infected RBCs (Figures 5A and 5B). The *SICAvar* transcript corresponding to the most abundantly expressed SICA protein in the Pk1(A+) clone (PkH_072710/PkH_072700), was only detected in the Pk1(A+) RNA, and predominantly in the ring stage compared to the trophozoite stage. A PkH_072700 specific riboprobe representing 409 bp of exon 2 hybridized to two prominent bands, both well over 10 kb. The largest transcript is most abundant and it is also evident as a distinct band in the trophozoite stage. A second comparable membrane was hybridized to a specific riboprobe representing 555 bp of exon 2 from PkH_051981 [31]. This *SICAvar* gene ID corresponds to the first two exons encoding the 205 kDa SICA protein shown here and in published studies to be predominantly expressed in the Pk1(B+)1+ infected RBCs. By northern blot, it is clear that this transcript is highly expressed and maintained as full length in the Pk1(B+)1+ parasites, but not in the Pk1(A+), Pk1(A-)1- and Pk1(B-)1- parasites. Smaller RNA signals could represent partial transcripts, differentially transcribed, spliced or processed *SICAvar* mRNA, or RNA made *de novo*; further investigation will be required to discern the options. The riboprobes were carefully selected for these experiments to be gene specific. 

### Quantitative RT-PCR corroborates unique patterns of *SICAvar* expression in Pk1(A+) and Pk1(B+)1+ infected RBCs

**Figure 6:**
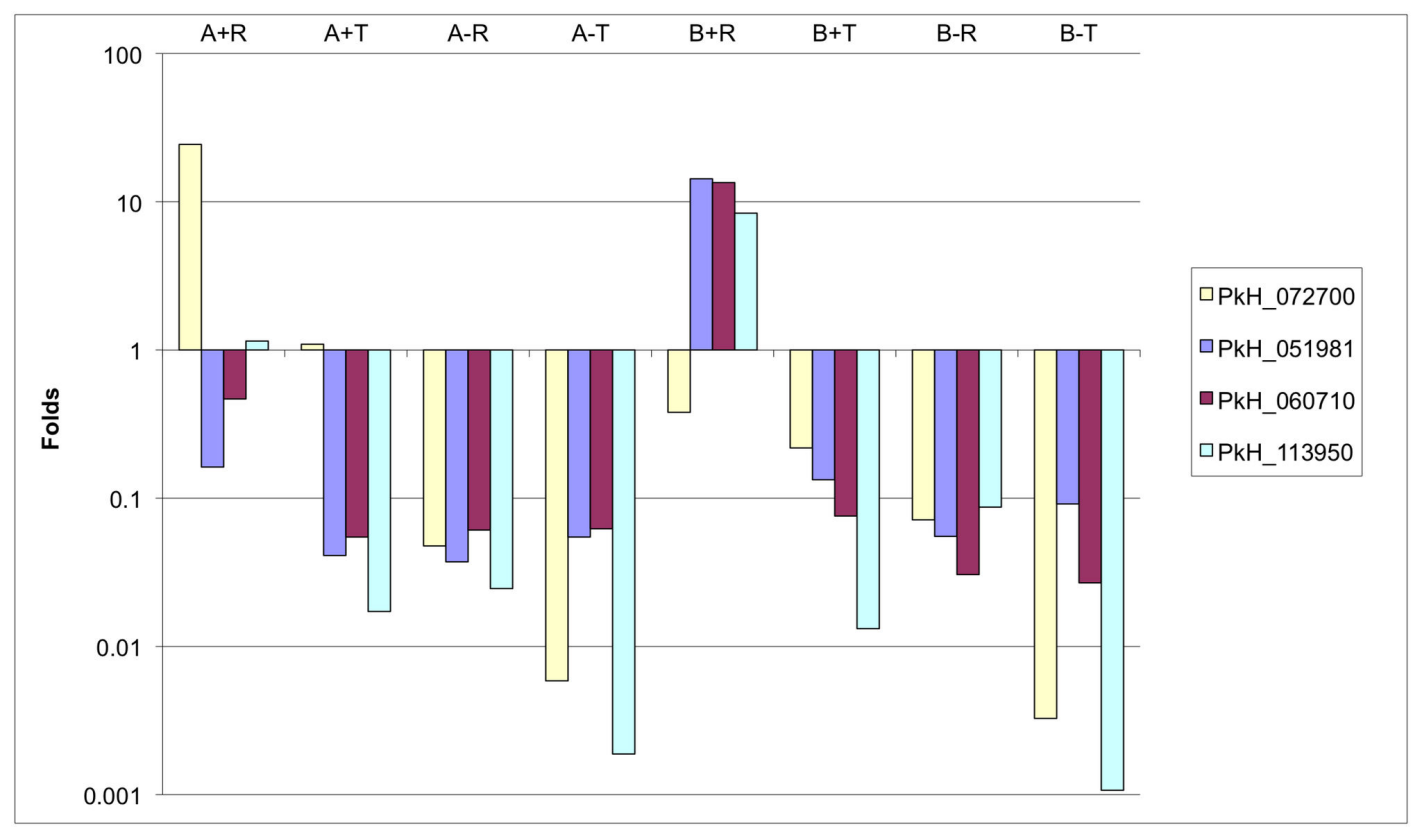
Quantitative Real Time-PCR profiles of specific *SICAvar* transcripts in Pk1(A+), Pk1(B+)1+ and the corresponding [SICA- **] parasite from ring (R) and trophozoite (T) stages**. The relative transcript amounts corroborate the northern blot data shown in Figure 5. Amplicons represent gene-specific regions of the most predominant proteins detected by LC-MS/MS from Pk1(A+) (PkH_072700) and Pk1(B+)1+ parasites (PkH_051981, PkH_060710 and PkH_113950), respectively.

We used qRT-PCR to examine the relative abundance of transcripts representing the major *SICAvar* genes identified by LC-MS/MS analysis in Pk1(A+), Pk1(A-)1-, Pk1(B+)1+ and Pk1(B-)1- ring and trophozoite-stage RNA samples (Figure 6). Gene-specific primers and MGB probes were used with pooled cDNA as an external control and the results were normalized to the *seryl tRNA synthetase* housekeeping gene. The resulting profiles and conclusions in essence mirror the transcription data shown in the northern blot experiments (Figure 5). The *SICAvar* gene represented by PkH_072700, which was predominantly identified from LC-MS/MS analysis of Pk1(A+) expressed SICA proteins was shown by qRT-PCR to be highly represented only in this clone, and specifically in the ring stage as expected. These transcript levels were measured by the amplification of 124, 84 and 127 bp sequences, respectively from exon 2 of PkH_072700, PkH_051981 and PkH_060710 sequences (Table S1); these are situated in the same region as the riboprobes used in the northern blots. The *205B SICAvar* allele transcript was highly represented only in the Pk1(B+)1+ parasites, and specifically the ring-stage. The genes encoding the next most abundant SICA antigens found in Pk1(B+)1+ parasites, represented by gene IDs PkH_060710 and PkH_113950, were likewise proportionally highly represented in these experiments. 

## Discussion

This research for the first time provides evidence at the molecular level supporting the spleen’s remarkable role in the regulation of variant antigen gene expression in malaria infections. The evidence is clear when one investigates *P. knowlesi SICAvar* transcripts from SICA[+] parasites that had been grown in intact rhesus monkeys in comparison with those from SICA[-] parasites generated in splenectomized animals. . Critically, there is an obvious abundance of full-length *SICAvar* transcripts detected by northern blot in the SICA[+] samples, and the complete lack of such signals in SICA[-] samples. On first appearances, these data could suggest that transcription of *SICAvar* genes is completely silenced in the splenectomized host. An alternative interpretation could be that *SICAvar* transcripts can be produced in the SICA[-] parasites but they become rapidly processed or degraded. Stage-specific qRT-PCR experiments in fact suggest that such processing may be occurring, as a low-level of 5’ and 3’ designed transcript amplicons can be detected by qRT-PCR in SICA[-] RNA; the presence of putative specific regulatory non-coding RNAs may also explain these results. Notably, such *SICAvar* products were detected at significantly lower levels in the Pk1(A-)1- and Pk1(B-)1- RNA samples than in their SICA[+] counterparts. The level detected in the SICA[-] ring-stage RNA was in fact comparable to that found in SICA[+] trophozoite RNA, which is always minimal in comparison and only weakly visible on northern blots compared to strong SICA[+] ring-stage signals (Figure 5) and [29]. The transcriptional origin and nature of these SICA[-] RNA segments remain to be explored in detail. This will be possible in the near future with the availability of an updated *P. knowlesi* genome sequence database with an improved assembly of the *SICAvar* genes and stage-specific RNAseq data (unpublished data). We have similarly observed a major reduction in *SICAvar* transcript signals in global microarray profiles from parasites grown in splenectomized animals or *in vitro* culture (unpublished data). 

The absence of full-length *SICAvar* signals on northern blots with SICA[-] ring-stage samples is likely to be attributed to some degree of transcriptional repression in the SICA[-] parasites, and may involve epigenetic mechanisms comparable to those being discovered with regards to *var* gene regulation in *P. falciparum* [2,39–41]. Importantly, our data supports the proposition that the spleen (and possibly other host factors working in synergy) is critical for *SICAvar* transcription, and, we predict, the accumulation and maintenance of full-length *SICAvar* transcripts targeted for translation. qRT-PCR data confirming the presence (though vastly reduced amounts) of PCR-targeted transcript segments, suggests multifactorial synergistic and well-honed molecular processes. We predict that transcriptional control mechanisms as well as post-transcriptional gene-silencing tactics may result in the stage-specific maintenance of the low level of *SICAvar* transcript sequences that are detectable by qRT-PCR in the absence of the spleen. 

The natural turnover of RNA observed in the *P. knowlesi* intra-erythrocytic development cycle (IDC) from SICA[+] parasites does not simply result in degraded RNA. This point is illustrated by the presence of as many as 22 *SICAvar* transcripts (corresponding to the last few exons) represented in a Pk1(B+)1+ cDNA library produced with RNA from late trophozoite and early schizont-stage parasites.(Figure S2) and [29],[30]. It is noteworthy that this set of cDNAs did not have a predominant sequence, and in fact, only four corresponded to the *205 SICAvar* allele known to produce protein in the Pk1(B+)1+ parasites. Furthermore, the putative products encoded by the majority of these cDNAs were not identified via gene ID hits in the LC-MS/MS experiments reported here in Figure 5. These *SICAvar* gene transcripts clearly were not destined to be translated. Hints of translational repression have also been revealed in principle by evidence that *SICAvar* transcripts detected by microarrays are not necessarily expressed as protein (unpublished data). As postulated earlier for SICA[+] parasites [30], it appears that some form of *SICAvar* RNA post-transcriptional gene silencing mechanism(s) may be operating in both SICA[+] and SICA[-] parasites to achieve translational repression. Additional research is warranted in this area. Translational repression has been described for the *P. falciparum var2csa* gene [42,43] and in *Plasmodium* sexual stage biology [44,45]. This field remains a relatively new area of exploration in *Plasmodium* parasites and malaria infections. A high degree of mRNA turnover in *P. falciparum* has also been noted on a genome-wide scale, especially in the early intra-erythrocytic cycle [46].

We suspect that both processed *SICAvar* mRNA and putative *SICAvar* non-coding transcript sequences are being detected in our RT-PCR experiments and on northern blots. Partial var gene sense and antisense non-coding RNAs have been identified in *P. falciparum* and confirmed to be products of a bi-directional promoter in the *var* gene intron [36,47-49]. Whether a functional promoter(s) exists in *SICAvar* genes within their comparable ‘final’ intron or within other introns, remains to be determined, reviewed in 1. It is worth noting in this regard that the final intron has conserved motifs within the *SICAvar* gene family. Sense and antisense non-coding RNAs are now known to be common in *P. falciparum* [50-52]. Their functions remain largely unexplored but they are predicted to be important for gene regulation.

Importantly, our data raise new questions regarding the molecular processes relating to variant antigen gene expression in a living host (with or without a spleen) compared to *in vitro* culture environments. Some splenic and potentially other host factors may be synergistically important for the natural *in vivo* regulation of the *SICAvar* gene family. Similar factors may also apply for *var* gene expression. In support of this possibility, cytoadherence of *P. falciparum* iRBCs (attributed to PfEMP1 expression [15,53]) was shown to be reduced in *Saimiri sciureus* monkey hosts lacking a spleen [23,24]. It will be of interest to revisit such non-human primate studies at the molecular level and compare *var* gene expression profiles in monkeys with and without the spleen present. We would predict a similar outcome as shown here for *SICAvar* expression: full-length *var* transcripts evident on northern blots from intact monkeys but not after passage in splenectomized animals. Such studies would also be valuable with *P. coatneyi*, a simian malaria parasite that is closely related to *P. knowlesi* [54], but which expresses ‘knobs’ and characteristics of cytoadherence and deep vascular sequestration like *P. falciparum*, reviewed in 1,20,55,56. *Plasmodium coatneyi* has a large multigene family with multi-exon structural features similar to the *SICAvar* genes and may be similarly regulated in macaques. *Plasmodium fragile* is likely to have a similar family, based on prior knowledge of antigenic variation in this related simian malaria species [57,58]. The strategic use and comparison of these various *in vivo* non-human primate models may facilitate our understanding of *P. falciparum* *var* gene expression, regulation, and pathogenesis. Interestingly, preliminary evidence is also materializing to show that placental tissue may be important to regulate the expression of *P. falciparum* *var* genes that are expressed during pregnancy [43].

In light of our data showing many transcript sequences are produced in SICA[+] parasites, without all of them necessarily being translated, the data of Wang et al. 2009 is intriguing [59]. This group has reported the detection of *var* transcripts representing 90% of the *var* gene family in the blood of a malaria naive vaccine trial volunteer who had been infected with *P. falciparum*, NF54 strain sporozoites [59]. This study and its interpretation could appear contrary to the basic tenet of antigenic variation and immune evasion; i.e., that mechanisms are in place to prevent the expression of all but one variant protein from the family at the same time. Wang and others suggested that the parasite population may express most or all *var* genes early on in an infection and that selection defined by cytoadherence to receptors or adhesion-blocking antibodies may then take place to ultimately result in the mutually exclusive expression of specific *var* types [59]. It remains unclear how such selection processes would become limited to the expression of one (or a few) PfEMP1 when the family shares various combinations of cysteine-rich domains, many with common adhesive characteristics, reviewed in 60. It also remains unknown how switches would then occur over time to develop chronic infections, characteristic of the process of antigenic variation and predicted to involve both inducing and opsonizing antibodies [6,7,9]. Based on the original studies and ongoing data coming from the *P. knowlesi* model system, we again put forth the alternative and potentially complementary view that most if not all *SICAvar* genes are transcribed (in some form) and certain genes become upregulated in the host blood-stage infection, as a rule (dependent upon some splenic factor), but that the majority of the transcripts are then silenced, leaving only those that are destined to be translated as intact messages [1,30]. This possibility, which would prevent the rapid expression and exposure of the variant family to the immune system, had been proposed by Piet Borst as one option for how *P. falciparum* may control expression of the *var* multigene family and its proteins [61]. With this scenario, variant type switches would remain the outcome of an antibody induced process, along with splenic factors that determine the state of gene expression. More recent reviews by Piet Borst reflect upon various mechanistic possibilities and many questions still in need of answers for *Plasmodium* and other organisms [62,63].

The majority of studies on the mechanisms of *P. falciparum* *var* gene expression, carried out since the discovery of the *var* gene family in 1995 [36-38], have been based on *in vitro* work, reviewed in 39,64,65. While clearly challenging for *P. falciparum* studies, the importance of studying antigenic variation in the context of the host environment has been stressed [1,66] and an increasing number of studies over the last decade have aimed to compare, contrast, and model results generated from *in vivo* or *ex vivo* iRBC samples acquired from human *P. falciparum* infections [26,59,66-75]. Such studies are also beginning to recognize the potential impact on *var* gene expression and switching depending on whether individuals are naïve and experiencing a more severe case of malaria or have previous exposure to the parasite and some degree of adaptive immunity. 

We provide evidence for the first time that SICA[-] parasites do not produce SICA proteins, consistent with the lack of detectable full-length *SICAvar* transcripts in SICA[-] parasite RNA. Most telling, we have used sensitive LC-MS/MS proteomic methods to confirm that SICA protein is not present in SICA[-] trophozoite-infected RBCs. It is known that when cloned SICA[-] parasites are reinoculated into an intact rhesus monkey SICA expression at the surface of the iRBCs can be reinstated [9]. Future directions include investigating what SICA proteins become expressed under these ‘reactivation circumstances’ or after passage through mosquitoes and defining the mechanistic processes and controls. Another major finding presented here is that the SICA antigenic switch from the Pk1(A+) phenotype to the Pk1(B+)1+ phenotype is ‘complete’, in that none of the SICA proteins detected in the parent clone are detected in the progeny clone. The complete change in protein expression is quite impressive. This switch can be defined as a dramatic shift in expression and not the result of the progression and selection of iRBCs that have drifted in their expression characteristics.

Among the *SICAvar* gene IDs detected by LC-MS/MS, curiously, PkH_040630 (detected with two unique peptides) represents an isolated 2-exon sequence with a PEXEL motif and start and stop codons. The PkH_040630 sequence is immediately upstream from PkH_040620 on the antisense strand. This small sequence lacks the majority of a typical SICA protein including the C-terminal cytoplasmic domain that would be recognized by the pan-SICAcyto antiserum used for the immunoprecipitation reactions, but it may have been pulled down in a complex. Another possibility is that PkH_040630 may have been alternatively spliced to *SICAvar* exons downstream of PkH_040620. The published sequence downstream, however, is truncated by a gap, leaving this an open question.

We have presented a compendium of expressed *SICAvar* genes and proteins for the first time to define the classic Pk1(A+) and Pk1(B+)1+ cloned populations. These data reveal that these parasites cannot be strictly defined by two SICA proteins representing the high molecular weight protein doublets originally observed by SDS-PAGE [8]. Nevertheless, they continue to express specific predominant SICA proteins, and in particular the originally defined 205 kDa SICA protein in the Pk1(B+)1+ parasites, even after many passages over time through intact rhesus monkeys; thus, overall, these clones can be viewed as stable with regards to their SICA expression [29-31]. It remains unknown whether each of the proteins detected by LC-MS/MS is actually expressed on the surface of the respective infected RBCs. Each of the SICA proteins was identified by unique peptides, thus we are confident they were expressed, but there is presently no evidence of the transport of every protein to the surface of the infected host cells. Such investigations will require the development of specific non-crossreacting antibody reagents, which are a challenge to produce for members of the SICA family. Attempts at expressing specific recombinant SICA proteins to develop specific antibodies and study the expression at a cellular and population level have been complicated by 1) the high degree of conserved sequences across the family and 2) the facts that SICA recombinant proteins tend to be insoluble and may not represent the natural conformation of the antigens. Proteomic and cell biological analyses of *P. falciparum* parasites have shown that multiple EMP1 variant antigens can be detected on the surface of infected erythrocytes [60,76,77]. In addition, a transgenic system expressing truncated PfEMP1 proteins showed that more than one PfEMP1 could be exported to the RBC surface. Thus, there does not seem to be a mechanistic barrier preventing the expression of multiple variant antigens on infected cell membranes [78].

We envision multiple levels of genetic and epigenetic transcription and post-transcriptional control, which ultimately determine which if any *SICAvar* genes are transcribed and which are translated. The *SICAvar* genes/transcripts/protein expression may in fact be in an ‘off state’ prior to entering a vertebrate host, as shown to be the case in sporozoites for the *var* genes [79], and the *in vivo* blood-stage environment including passage through the spleen (or factors therefrom) may provide the epigenetic stimuli needed to permit their coordinated/regulated expression. We speculate that in splenectomized animals, such predicted ‘on signals’ are missing. In line with a recent report showing the activation of all *var* genes in the absence of the *PfSETvs* gene [77], epigenetic control involving methylation of histones for silencing and demethylation for activation may likewise be paramount and should be investigated.

On a population level, a few important questions remain. It remains possible that individual parasite-infected RBCs are able to express a set of major and minor SICA proteins, or that the SICA[+] clonal populations being studied have undergone some degree of drift with the vast majority expressing the predominant proteins and a relative few expressing the minor proteins. Similarly, SICA[-] clones may represent a population of parasites that are all expressing a low level of transcripts that are not destined for translation, or a vast majority that are entirely in the off state and a relative few in the on state. These reflect some of the critical questions relating to *SICAvar* gene regulation and switching of variant phenotypes that can now be addressed in *P. knowlesi* by investigating epigenetic mechanisms and using quantitative imaging technologies.

## Materials and Methods

### Ethics Statement

All animal experiments in the current study were conducted in AAALAC-accredited facilities at the Yerkes National Primate Research Center in accordance with the Animal Welfare Act and the Guide for the Care and Use of Laboratory Animals. Emory University’s Animal Care and Use Committee (YER-259-2007) approved all experimental protocols. Temperatures in animal areas are controlled and monitored by an automated on-line system. The system has set parameters for high and low temperatures, maintained at 72± 5F. The photoperiod is set at a 12/12 cycle. Rhesus macaques are housed singly, and provided with one of the following: forage board, peanut feeder, challenger ball, or other similar device for enrichment. In addition, destructible enrichment is distributed as prescribed to monkeys. Rhesus macaques receive jumbo biscuits (15% protein). Animals are fed twice daily. The amount of food is adjusted depending on species, sex, age, weight and specific number of animals living in a group. Consumption is monitored and adjustments are made as necessary so that animals get enough food with minimum waste. Enrichment produce is fed twice weekly. All animals are monitored daily by veterinary staff for potential health problems. Experienced personnel trained all animals to voluntary present at the front of the cage for skin prick using positive reinforcement. For large blood collection volumes, animals were sedated with Ketamine (dose/route) or Telazol (dose/route) to minimize distress. No animals were sacrificed in this study.

### Genome Data

Version 5.2 of PlasmoDB [80] was available at the time of proteomics analysis so SEQUEST searches against NCBI at that time would have been representative of this version. Initial BLAST analyses cited here involved all subsequent versions of PlasmoDB up the present, but all were repeated with the latest version, 9.3. PlasmoDB was also used to generate Figure S1, the chromosomal location of identified sequence IDs. Notably, annotation of the *P. knowlesi SICAvar* gene family has remained essentially unchanged since the publication of the genome [32]. 

### Parasite material and antisera

The *P. knowlesi* clonal parasites, Pk1(A+), Pk1(A-)1-, Pk1(B+)1+ and Pk1(B-)1- were expanded in rhesus macaques as described [9]. SICA [+] parasites were expanded in naïve rhesus macaques, and SICA [-] clones were expanded in splenectomized animals [22]. After achieving sufficient parasitemias, whole blood was drawn at no more than 10 ml/kg host weight. Platelets and leukocytes were removed by treatment with 250 µg/ml of ADP (adenosine di-phosphate, Sigma, St. Louis, MO, USA) for 5 min at room temperature, followed by passage through a packed column of glass beads and a Plasmodipur filter. Cultures were started in pre-warmed RPMI 1640 supplemented with 30 mM HEPES, 28.5 mM NaHCO_3_, 2 g/L glucose, 10 mg/L hypoxanthine, 25 mg/L gentamicin, and 10% human AB+ serum. 

Parasites were maintained at 37°C through the schizont stage of one life cycle. Ring stage parasites were removed after 2 hours in culture, and subsequent stages were taken every four hours through the evening and following morning. Parasites at the late trophozoite stage were purified on a 52% percoll gradient, washed 3 times with incomplete RPMI 1640, resuspended in SDS sample buffer, and stored at -80°C. 

Total RNA was extracted with Trizol LS (Invitrogen, Carlsbad, California, United States) as described [31], followed by DNase I digest and purification with the RNeasy Midi kit (Qiagen, Valencia, CA, USA). RNA was stored in 70% ETOH and RNase-free water at -80°C in 30 µg aliquots. When needed, 1/38.5 volume sodium acetate pH 5.2 was added and the sample was incubated for 2 hours at -80°C before centrifuging at max speed 4°C. The pelleted RNA was washed once with 75% ETOH, dried, and resuspended in RNase-free water at approximately 1 µg/ml. RNA quality and concentration was determined using a Nanodrop spectrophotometer. 

A pan-SICACyto antiserum was generated commercially in rabbits (Covance) by inoculating a recombinant protein (SICACyto) representing the conserved C-terminal cytoplasmic domain. A BLAST search with this region from the Pk1(A+) and Pk1(B+)1+ *SICAvar* genes hit 80 *SICAvar* genes with greater than 85% identity at the protein level. The primers used were For 5’- ATTCCCGGGTTTCTGCTATGGCTTATT-3’ and Rev 5’- ATTGCGGCCGCCGGAACCTAAATTTGGAA-3’. This was cloned into the pGEX-4T-3 expression vector and amplified in BL-21 star cells using 0.1 mM IPTG. Inclusion bodies were isolated and purified using 1% Triton-X 100, then solubilized in 10M urea in PBS. The solubilized protein was diluted into a 50X volume of refolding buffer (44 mM L-arginine, 55 mM Tris, 21 mM NaCl, 0.88 mM KCl, pH 8.2) and incubated for 24 hours at 4°C. The refolded protein was concentrated with an Amicon concentrator unit, model 8400, followed by exchanges the buffer to 1X PBS. Concentrated protein was aliquoted and stored at -20°C or -80°C for further use. The Cyto17 SICA antiserum was produced previously [29].

### Metabolic labeling of parasites, immunoprecipitations, SDS-PAGE analysis

*Plasmodium knowlesi* parasites were metabolically labeled in methionine/cysteine-deficient RPMI-1640 (ICN Biomedicals, MP Biochemicals, Santa Ana, CA, USA) with 10% human AB serum, using 50 µCi of trans-[35S]-methionine/cysteine (ICN Biomedicals) per ml of medium and 10% v/v regular RPMI-1640 added. Metabolically labeled parasites were extracted with a mixture of 1X NET (150 mM NaCl, 5mM EDTA, 50 mM Tris base, pH 7.4), 1% NP-40 and a protease inhibitor cocktail (10 mM EDTA-Na2; 1mM PMSF; 0.1 mM each of TPCK, TLCK, leupeptin, chymostatin, antipain and 3,4-DCI; 10 μM EP-64 and 1 μM pepstatin A) (Sigma). Detergent extracts were incubated with either the pan-SICACyto or Cyto 17 antiserum overnight at 4°C. The antibody/extract complex was then immunoprecipitated by adding either goat anti-mouse IgG-conjugated sepharose CL4B beads (GIBCO, Carlsbad, CA, USA) or protein A-sepharose beads for 2 hours with mixing at 4°C. Washes were performed twice with 1XNETT (150 mM NaCl/ 5 mM EDTA/ 50 mM Tris/ 0.5 % Triton X-100) buffer, twice with NETT/0.5M NaCl and a final wash with NETT/0.05% SDS. The beads were resuspended in SDS PAGE sample buffer, and then electrophoresed on 4-15% gradient SDS-PAGE gels. 

### Preparation of membrane ghosts

The infected RBC membranes, including their parasite-encoded proteins, were purified from the parasite ‘body’ using ficoll lysis/density gradients as previously described [81]. Briefly, Percoll purified infected RBCs were washed 2X in incomplete RPMI and a final wash in phosphate-buffered saline containing protease inhibitors. Infected RBCs were layered and centrifuged on a 5% (hypotonic), 7.5% and 15% ficoll separation gradient. Prepared membrane ghosts were lysed in sample buffer and electrophoresed on 4-15% gradient SDS-PAGE gels. 

### Immunoprecipitations and proteomics

Immunoprecipitations of Pk1(A+) and Pk1(B+)1+ iRBC extracts were prepared as described [82] using the pan-SICACyto antibody that is pan-reactive to the conserved cytoplasmic domain of expressed SICA proteins. Briefly NP-40/SDS extracts were incubated with antibody conjugated Protein G beads overnight at 4°C, washed, resuspended in SDS-PAGE loading buffer and resolved 4-20% gradient gels (Bio-Rad). 

### Mass spectrometry analysis

Peptides were generated as previously described [34]. Briefly, protein bands were excised from SDS-PAGE gels and gel pieces destained and dried. Dried gel bands were digested at 37°C overnight with 0.4g of proteomics grade trypsin (Sigma, St. Louis, MO, USA). The resulting peptides were then extracted with 0.1% trifluoroacetic acid in 50% acetonitrile (Sigma), desalted and concentrated using ZipTip pipette tips containing C18 reversed-phase media (Millipore, Billerica, MA), and then washed in 0.1% trifluoroacetic acid and eluted in 0.1% trifluoroacetic acid/50% acetonitrile (Sigma). Cleaned peptides were analysed by reverse-phase LC-MS/MS [83] using an LTQ-Orbitrap mass spectrometer (Thermo Finnigan). A reverse database strategy using the SEQUEST algorithm was implemented to evaluate false discovery rate; the matched peptides were filtered according to matching scores to remove all false matches from the reverse database [84]. Only proteins that were matched by at least two peptides were accepted to further improve the confidence of identification. The peptides were then searched against the NCBI database, with searches being limited to *Plasmodium* results. The *P. knowlesi* identified protein sequences were then used to BLAST (Basic Local Alignment Search Tool) against the NCBI database for sequence homologies.

### Northern blots

For oligonucleotide-probed northern blots, 20 μg of total RNA was electrophoresed and transferred to a positively charged nylon membrane [85]. The membrane was UV cross-linked and baked for 2 hours at 80°C with vacuum, and then prehybridized four hours at 42°C with SuperHyb-Oligo (Ambion-Life Sciences). The membrane was hybridized overnight at 42°C with 1x10^6^ cpm/ml [α-32P]-labeled probe (Oligonucleotide 3’ End Labeling Kit, NEN-Perkin Elmer). Washes consisted of two 30 min washes with 2X SSC, 0.1% at 42°C, and then with 1X SSC, 0.1 % SDS at 42 °C until the background signal was low. 

Northern blot experiments were conducted essentially as previously described [31]. Specific riboprobes were generated by PCR-amplifying specific 5’ regions of the PkH_051981 and PkH_072700 *SICAvar* genes (400-550 bp in length, Table S1), which in general are more unique than downstream sequence. BLAST searches were done to ensure the most unique primers and amplicon possible for each gene. The DNA was purified through Qiagen spin columns and shown to be one band by gel electrophoresis. The T7 promoter sequence was added to reverse primers, and used to *in vitro* transcribe antisense riboprobes from 1 µg PCR product, using the MaxiScript kit (Applied Biosystems), alpha ^32^P UTP, 800 Ci/mmol specific activity, incubated at 37°C for 15-30 min. Unincorporated nucleotides were removed with NucAway G50 columns (Applied Biosystems) before being added to 10 ml Ultrahyb (Applied Biosystems) buffer pre-warmed to 65°C. Blots were prehybridized with Ultrahyb buffer for a minimum of one hour at 65°C, the buffer removed, and fresh buffer with the riboprobe was added and incubated overnight at 65°C in a hybridization bag. Generally 2 low stringency washes of 2X SSC/0.1% SDS were done at 65°C, 30 min each, followed by 2 high stringency washes of 0.1X SSC/0.1% SDS at 65°C, 30 min each. Blots were rinsed with 2X SSC before exposure to MS film (Kodak, Rochester, NY, USA). Exposures were taken at 3 hours, overnight, and at 72-96 hours depending on the intensity of signal. 

### Quantitative RT-PCR

For absolute quantification of the 205B gene in Pk1(B+)1+ and Pk1(B-)1- the following protocol was used. Plasmid controls were generated by cloning the products of rt-pcr reactions from Pk1(B+)1+ parasites into the pCRII-TOPO^®^ vector (Invitrogen) and transforming into TOP10 cells. The PCR product represented sequence of exon 10 through the block III of the 3’UTR of the *205B SICAvar* allele [29]. Positive clones were screened and sequenced in both directions to confirm fidelity and orientation of the insert. Specific primers and a MGB Taqman probe were designed using Primer Express software (ABI; forward, 5’- ATGGAATACCATCCTGGGATAC-3’, which starts at the end of exon 11 and crosses the junction into exon 12, and reverse, 5’-GTTCAACTTCTTTCTGCTTATTTCC-3, probe, 5’-6FAM TGAGCACTGAAATCGA MGBNFQ-3’).

The quality of all RNA samples was tested using the RNA 6000 Nano Assay on an Agilent bioanalyzer. One µg total RNA from ring and trophozoite stages of Pk1(B+)1+ and Pk1(B-)1- parasites was used in each reverse transcription reaction (Thermoscript, Invitrogen ). The following primers were used: Cyto5, a conserved reverse primer in exon 12; VC27, a forward primer in exon 10; and oligo(dT). The RT reaction ran for 90 minutes at 55°C. The cycling conditions included an initial 10 min denaturation at 95°C, followed by 40 cycles of 95°C 15s, and 60°C 1 min (ABI 7500). Standard curves of Pk1(B+)1+ controls from 1x10^8^ to 1x10^2^ copies were used to determine copy number. The data were normalized by including *seryl tRNA synthetase* as an internal control using the following primers and MGB probe set: forward, 5’-GGTGGCGGCGCAAA-3’, reverse, 5’-CGATGCCCTCACCATTCTG-3’, probe, 5’-6FAM CGAGGAGACAACAGAGG MGBNFQ-3’. We checked this housekeeping gene by northern blot to confirm that it is constitutively expressed from ring to late trophozoites (data not shown). 

To determine the relative expression of major transcripts in the Pk1(A+) and Pk1(B+)1+ clonal populations, specific primers and MGB probes were designed for the *SICAvar* genes, PkH_051981, PkH_060710, PkH_072710, PkH_113950, as well as the housekeeping gene, *seryl tRNA synthetase* (Table S1). For each, extensive BLAST searches were done to identify the most unique region. Either one primer or the probe for each gene was designed to cross an exon junction in order to eliminate possible amplification of remnant gDNA in the RNA preps. Each primer set was validated in terms of PCR efficiency in order to accurately calculate relative quantification by the ΔΔCt method. cDNA was synthesized from 1 µg total RNA of each clone, ring and trophozoite stage, with Thermoscript enzyme (Invitrogen) and random hexamers at 57°C for 90 min, and cleaned through a Qiagen quick spin column. The PCR was carried out on a 384-well optical plate (Applied Biosystems) in a 10-µl reaction volume containing with TaqMan Gene Expression Master Mix (Applied Biosystems), with primer concentrations of 50 pmol. All sequences were amplified using the Applied Biosystems 7900HT Sequence Detection System with the PCR profile: 50° for 2 min, 95°C for 10 min, followed by 45 cycles at 95°C for 15 s, and 60°C for 1 min. Samples were tested in duplicate, in parallel with the housekeeping gene *seryl tRNA synthetase*. A pooled cDNA sample was used as an external control. Real-Time StatMiner^TM^ software (Integromics, Inc., Madison, WI, USA) was used to perform a quality control for all runs and relative quantification ΔΔCt analysis to calculate the fold differences between samples.

## Supporting Information

Table S1 **Oligos for PCR, qRT-PCR, protein expression, and generation of riboprobes for Northern blot experiments.**
(XLSX)

Figure S1 **Chromosomal location of gene IDs identified by LC-MS/MS analysis of trophozoite extracts.** (A) Chromosomal location of genes and gene fragments identified by proteomics in Pk1(A+) and (B) Pk1(B+)1+ parasites. Blue indicates the gene is on the forward strand of DNA; red indicates the gene is on the reverse strand.
(TIF)

Figure S2 **Sequence IDs identified by updated BLAST searches of 22 clone sequences found in a previous Pk1(B+)1+ cDNA library screen** [30]. Sequence IDs in bold indicate peptide hits identified by LC-MS/MS of Pk1(B+)1+ trophozoite extracts.
(TIF)
