# Supplementary figures and images for: Spleen-Dependent Regulation of Antigenic Variation in Malaria Parasites: Plasmodium knowlesi SICAvar Expression Profiles in Splenic and Asplenic Hosts
Source: PLoS One. 2013 Oct 18;8(10):e78014. doi: 10.1371/journal.pone.0078014 (PMC3799730; doi:10.1371/journal.pone.0078014)

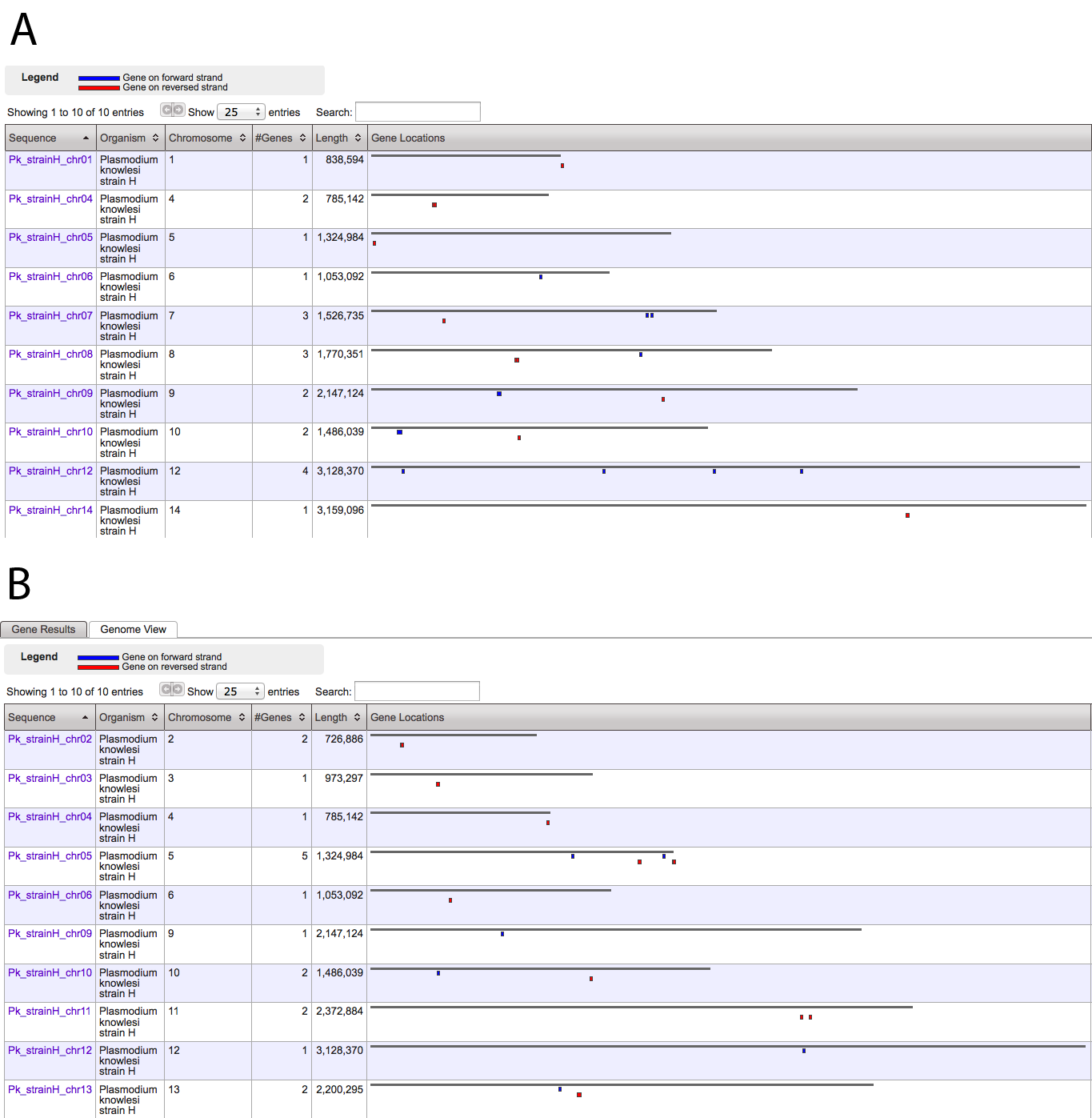

Supplement: Figure S1 — Chromosomal location of gene IDs identified by LC-MS/MS analysis of trophozoite extracts. (A) Chromosomal location of genes and gene fragments identified by proteomics in Pk1(A+) and (B) Pk1(B+)1+ parasites. Blue indicates the gene is on the forward strand of DNA; red indicates the gene is on the reverse strand. (TIF) [file pone.0078014.s002.tif]

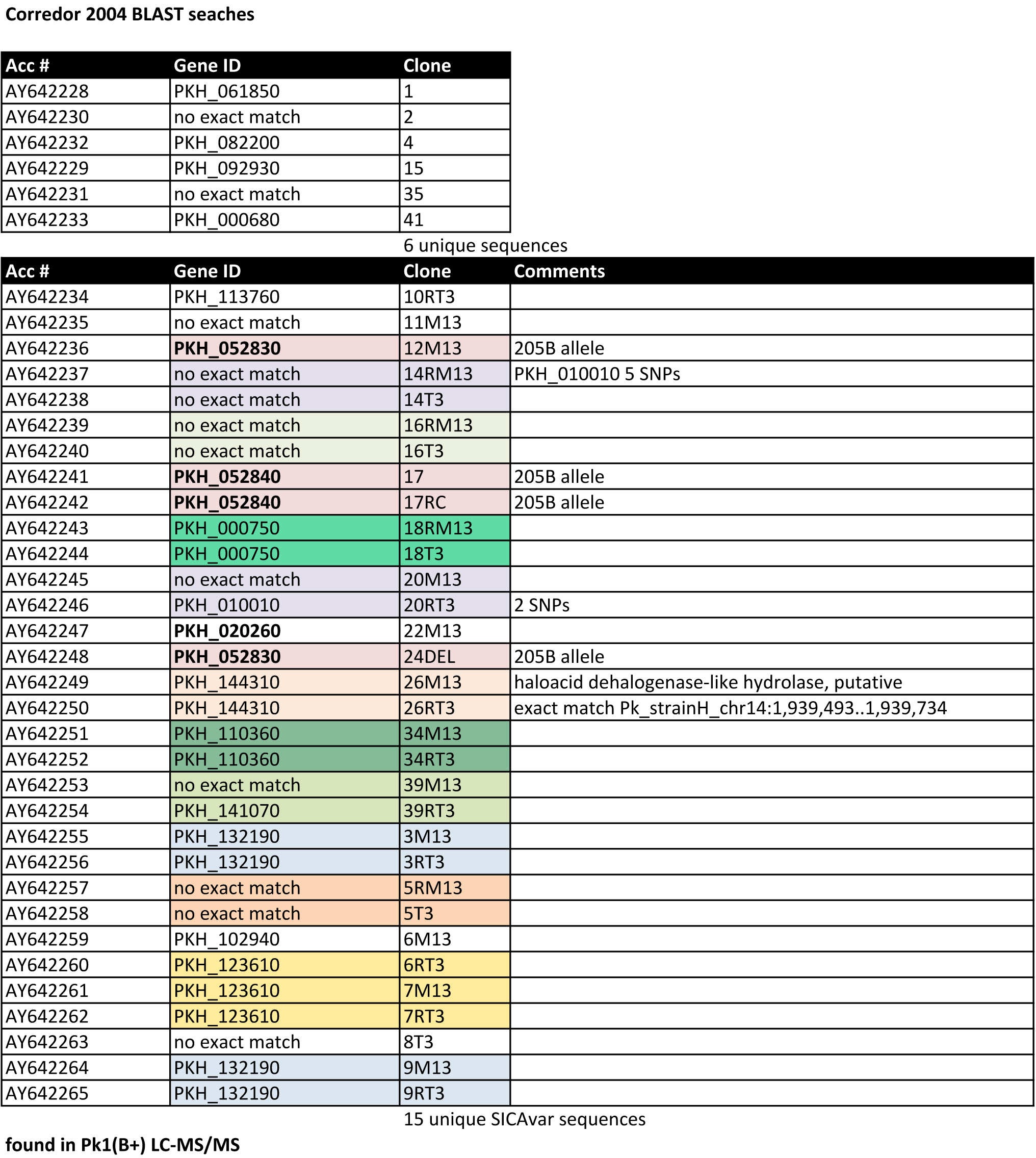

Supplement: Figure S2 — Sequence IDs identified by updated BLAST searches of 22 clone sequences found in a previous Pk1(B+)1+ cDNA library screen [30]. Sequence IDs in bold indicate peptide hits identified by LC-MS/MS of Pk1(B+)1+ trophozoite extracts. (TIF) [file pone.0078014.s003.tif]
